# Identification of Candidate Genes and Functional Pathways Associated with Body Size Traits in Hulunbuir Sheep Through GWAS Analysis

**DOI:** 10.3390/genes16040410

**Published:** 2025-03-31

**Authors:** Hengqian Yang, Tingting Li, Na Zhang, Jieran Chen, Yuting Zhang, Shiyu Peng, Lisheng Zhou, Runlin Ma, Zhichao Zhang, Qiuyue Liu, Haitao Wang, Jianning He

**Affiliations:** 1 College of Animal Science and Technology, Qingdao Agricultural University, Qingdao 266109, China; yhq17806253397@163.com (H.Y.); zls123668@qau.edu.cn (L.Z.); 2 State Key Laboratory of Molecular Developmental Biology, Institute of Genetics and Developmental Biology, Chinese Academy of Sciences, Beijing 100101, China; tingli0704@163.com (T.L.); zhangna4520@163.com (N.Z.); 17852401863@163.com (J.C.); zhangyut09@163.com (Y.Z.); pengsy@163.com (S.P.); rlma@genetics.ac.cn (R.M.); zhichaozhang0716@163.com (Z.Z.); qyliu@genetics.ac.cn (Q.L.)

**Keywords:** GWAS, candidate genes, body size, Hulunbuir sheep

## Abstract

(1) Background: The Hulunbuir sheep is a Chinese local sheep breed with good meat quality and exceptional cold-stress resilience. However, the growth performance of the Hulunbuir sheep is lower when compared to that of commercial breeds. Growth traits such as body weight and body size are critical, as they directly influence the meat production in sheep farming. Employing genome-wide association studies can identify SNPs and candidate genes related to growth traits in Hulunbuir sheep. (2) Methods: The chest girth (CG), cannon circumference (CC), hip width (HW), body height (BH), and body length (BL) of 799 Hulunbuir sheep were measured. All the animals were divided into three groups according to their age (Group 1: 0–6 months old, Group 2: 12–24 months old, Group 3: 48–70 months old). Subsequently, genotyping was carried out using the Sheep 40K liquid chip. A multi-trait genome-wide association study (MT-GWAS) was performed for each group of animals. (3) Results: A total of three candidate genes (*SLC9C1*, *VSTM2A*, *FRG1*) associated with body size traits were identified through GWAS analysis and KEGG pathway enrichment for Group 2. (4) Conclusions: This study identified three candidate genes related to body size in Hulunbuir sheep, providing genetic targets for marker-assisted selection (MAS) in Hulunbuir sheep.

## 1. Introduction

Sheep (*Ovis aries*) is a widespread farmed livestock all over the world. Serving as meat sources is one of the most vital roles of sheep in agriculture. Body size is a critical trait in sheep breeding for increasing meat production. It serves not only as a key indicator for the mutton sheep industry but also as a measure of sheep’s health status and feed conversion efficiency (FCR) [1,2]. Hulunbuir sheep, an indigenous breed native to Inner Mongolia in northern China, are renowned for their exceptional cold resistance, meat quality, and adaptability. Adult males of Hulunbuir sheep demonstrate an average body weight of 79 kg and body height of 72.6 cm, while females weigh 62.2 kg with an average body height of 67.8 cm. The average dressing percentage of adult Hulunbuir sheep is 50.4%, and the average net meat yield is 43.3%. Hulunbuir sheep reach sexual maturity at 5 to 7 months of age and are ready for initial mating at 18 months of age and have an annual average lambing rate of 113%. However, their lower productivity and slower growth performance compared to commercial breeds limit their farming potential. Genetic improvement through advanced breeding strategies can enhance the growth performance of Hulunbuir sheep.

Body size traits, as polygenic quantitative characteristics, significantly influence meat production efficiency in sheep breeding [3,4,5]. Body size measurements mainly include chest girth (CG), cannon circumference (CC), hip width (HW), body height (BH), body length (BL), etc., which have an important influence on meat production. The molecular markers that affect body size traits in sheep can be utilized in selective breeding. The application of genome-wide association studies (GWAS) has become increasingly common in the genetic analysis of complex traits. By combining SNP chips with GWAS, researchers can identify molecular markers linked to various economically significant phenotypes [6,7,8]. SNP chips with varying densities have enabled comprehensive coverage of SNP markers across the sheep genome, providing valuable support for conducting GWAS on economically important traits in sheep. Published GWAS studies in sheep have primarily focused on traits such as horn development, wool characteristics, milk production, growth and development, meat quality, reproduction, and disease resistance [9]. Multi-trait genome-wide association study (MT-GWAS) represents an advanced analytical framework that enables concurrent evaluation of multiple genetically correlated traits. Compared with traditional single-trait GWAS, MT-GWAS effectively exploits shared genetic architectures among phenotypic characteristics, which is a benefit for identifying pleiotropic associations [10,11].

Over the past few years, numerous studies have been conducted to identify the candidate genes affecting body size traits in sheep. A previous study indicated that two novel indels within the sheep *SIRT7* gene were related to rump width and chest depth [12]. Another study involving Ovine SNP50 whole-genome genotyping array identified genomic regions with high ROH frequency in 635 Chinese Merino sheep. This study highlighted *NCAPG/LCORL*, *FGF11*, and *TP53* as the candidate genes linked to body size [13]. In addition, 188 adult Qira black sheep were genotyped with a 630 K high density SNP chip. Six candidate genes associated with body size were identified through GWAS analysis. In these candidate genes, *ZNF704* was identified to be associated with body weight; *AK2* and *PARK2* were responsible for tail length; *MOCOS* and *ELP2* for chest width; and *MFAP1* for chest girth [14]. A GWAS on body size of Hu sheep identified 5 SNPs related to body height and 4 SNPs related to chest girth at the chromosomal significance level [15]. Based on the SNP data and growth traits of the Qira Black sheep and German Merino sheep, an association analysis identified 55 SNP loci, and there were 84 genes close to these SNPs [16]. Another GWAS on body size using Illumina high-density SNP chips involving 217 individuals indicated that 46 SNPs on 14 chromosomes were significantly associated with body size; genes such as *NCAPG*, *MSRB3*, and *HMGA2* were also identified in the vicinity of these SNPs and could be key candidate genes for body growth [17]. A series of candidate genes were identified by GWAS in 39 Tibetan sheep and 328 hybrids of Tibetan sheep and wild argali. The genes related to cell adhesion, angiogenesis, and gene expression regulation, such as *PCDH10* and *HMGN1*, were significantly identified to be associated with body weight and chest circumference. *MSRA*, *IQCH*, and *UBASH3B* were significantly associated with body height. The *BMRP1B* gene, associated with embryonic development, as well as bone and cartilage formation, was significantly correlated with body length. *MRS2* gene significantly correlated with hip height; *ACTR3B* and *DPP6* genes were significantly related to hip width; and *TBXT* was significantly related to tail length [18]. These potential molecular markers can be utilized for marker-assisted selection breeding to improve growth performance of sheep.

Major genes or SNP loci can be identified and used as molecular markers for sheep breeding. This study aimed to identify SNPs and candidate genes influencing body size traits (chest girth, cannon circumference, hip width, body height, body length) in Hulunbuir sheep through MT-GWAS, gene ontology, and KEGG pathway analysis. These newly discovered genetic markers are expected to serve as valuable references for molecular breeding within the sheep industry.

## 2. Materials and Methods

### 2.1. Experimental Animals

In this study, Hulunbuir sheep are supplied and bred by different farms located in the sheep breed conservation area of Evenk Autonomous Banner, a county of Hulunbuir city in Inner Mongolia Autonomous Region (License number BY-18072-01). Due to the significant age disparity, all the animals have been divided into three groups according to their age [19] (Table 1). The body size traits were measured after sheep shearing. All experiments were performed in strict compliance according to the guidelines of the Animal Advisory Committee at the Institute of Genetics and Developmental Biology, Chinese Academy of Sciences (approval No. AP2022015-C1).

### 2.2. Determination of Body Size Traits

The body size traits of Hulunbuir sheep were determined by referring to Technical Specifications for Sheep and Goat Stud Productivity Testing (NY/T 1236-2006). Measurements were recorded using specialized tools with animals standing naturally on flat surfaces. The CG is the circumference measured around the chest from the back end of the shoulder blades; the CC is the minimal horizontal circumference of the left front cannon bone; the HW is the distance between the two ischial tuberosities; the BH is the vertical distance from the highest point of the shoulder blades to the ground; the BL is the straight-line distance from the chest protuberance to the back end of the ischial tuberosity. The animals are grazed in a large field but not in one farm. Moreover, when measuring body size, the animals were held by an assistant to stand naturally and squarely on a firm and flat surface. 

### 2.3. Genotyping and Quality Control

In this study, 5 mL of venous blood was collected from each sheep and temporarily stored at −80 °C. DNA was extracted by (TIANamp Genomic DNA Kit, DP304, Tiangen, Beijing, China) according to the provided protocol. In brief, genomic DNA from blood sample was bound to a centrifugal column and washed by a unique buffer system provided in the kit. DNA was eluted by elution buffer and then stored at −20 °C. DNA samples from 799 sheep were genotyped using a Sheep 40K liquid chip [20]. Furthermore, PLINK v1.9 software [21] was used to conduct quality control with the follow principle: (1) SNPs with minimum allele frequency (MAF) < 0.05 were removed; (2) SNPs with the missing rate of SNP site (MISS) > 0.5 were removed; (3) SNPs of Hardy–Weinberg equilibrium (HWE) < 10^−6^ were removed. To ensure the quality of the data, we conducted a second round of filtering to confirm that call rates were at least 90% of the SNPs. After quality control, a total of 33,683 SNPs from Group 1; 37,282 SNPs from Group 2; and 37,883 SNPs from Group 3 were retained for subsequent analysis.

### 2.4. Principal Component Analysis

Genetic relatedness between individuals was assessed by performing principal component analysis (PCA) performed using GCTA software. In addition, the PCA results were visualized using PLINK software (version 1.9), with the horizontal and vertical axes representing different principal components.

### 2.5. Genome-Wide Association Study

Multi-trait genome-wide association study (MT-GWAS) was performed by GEMMA using the following multivariate mixed linear model for each of the three age groups; each group included five body size traits (CG, CC, HW, BH, and BL) [22]. The formula is as follows:

Y = WA + xβ^T^ + U + E; G ~ MN_n×d_(0, K, V_g_), E ~ MN_n×d_(0, I_n×n_, V_e_)

Y represents the n × d phenotype matrix composed of phenotypic data, n is the number of individuals, and d is the number of phenotypes; W = (w_1_, …, w_c_) is an n × c covariate matrix (fixed effects) including a column of 1s; A is a c × d matrix of the corresponding coefficients including the intercept; x represents an n-vector of genotypes; β is the effect sizes of the SNPs for the d traits; U is the random effects of n × d matrix; E is an n × d matrix of errors; K is the n × n relatedness matrix; I_n×n_ is a n × n identity matrix; V_g_ is a d × d symmetric matrix of genetic variance component; V_e_ is a d × d symmetric matrix of environmental variance component; and MN_n×d_(0, V_1_, V_2_) denotes the n × d matrix normal distribution with mean 0, row covariance matrix V_1_ (n × n), and column covariance matrix V_2_ (d × d). According to the software manual, V is a covariate. Then we calculated the association tests with multivariate linear mixed models using GEMMA (parameters: -bfile -k -lmm -o -n -c). We put the age (in months), gender, farm location, etc., in the file cov.txt, which is input by -c.

### 2.6. Gene Annotation

Based on National Center for Biotechnology Information databases (http://www.ncbi.nlm.nih.gov/, accessed on 7 March 2025, Ovis aries genome (Oar_v4.0 (GCF_000298735.2))) was used to identify genomic regions and candidate genes. Candidate genes for body size were searched within 20 kb of the upstream or downstream of significant SNPs, and genes functions were annotated.

### 2.7. Enrichment Analysis of the Candidate Genes

The *p*-values (*p* ≤ 0.05) of candidate genes significantly associated with body size traits were uploaded to the Database for Annotation, Visualization, and Integrated Discovery (DAVID) (http://david.ncifcrf.gov/list.jsp, accessed on 18 March 2025) for the Gene Ontology (GO) terms and Kyoto Encyclopedia of Genes and Genomes (KEGG) pathway analysis. 

## 3. Results

### 3.1. Descriptive Statistics

**Figure 1:**
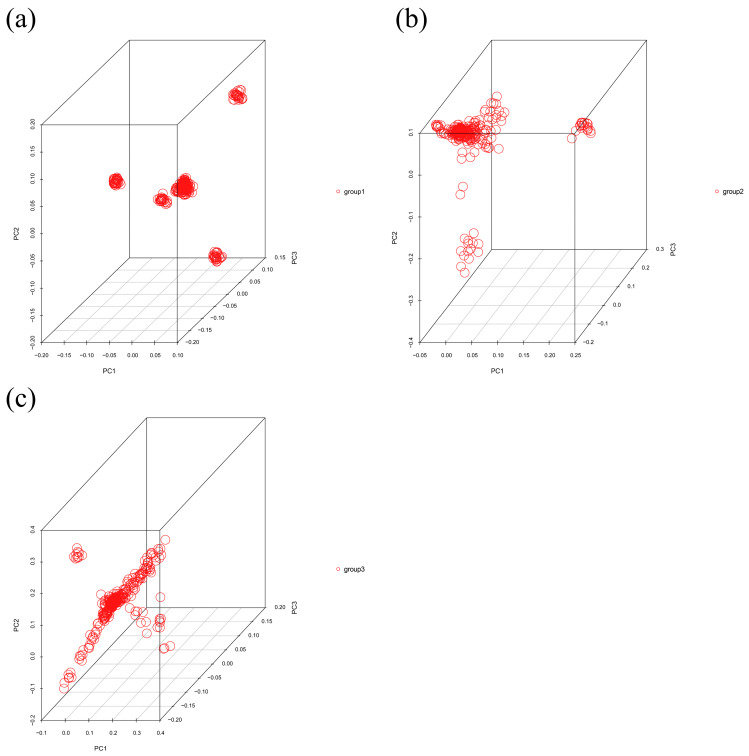
PCA result of population stratification of Hulunbuir sheep. (**a**) Group 1; (**b**) Group 2; (**c**) Group 3.

The descriptive statistics of body size traits (chest girth, cannon circumference, hip width, body height, body length), including mean and SD (standard deviation) values, are shown in Table 2. Based on the findings from the principal component analysis, it is evident that there is no obvious population stratification in the Hulunbuir sheep (Figure 1).

### 3.2. Genome-Wide Association Study

**Figure 2:**
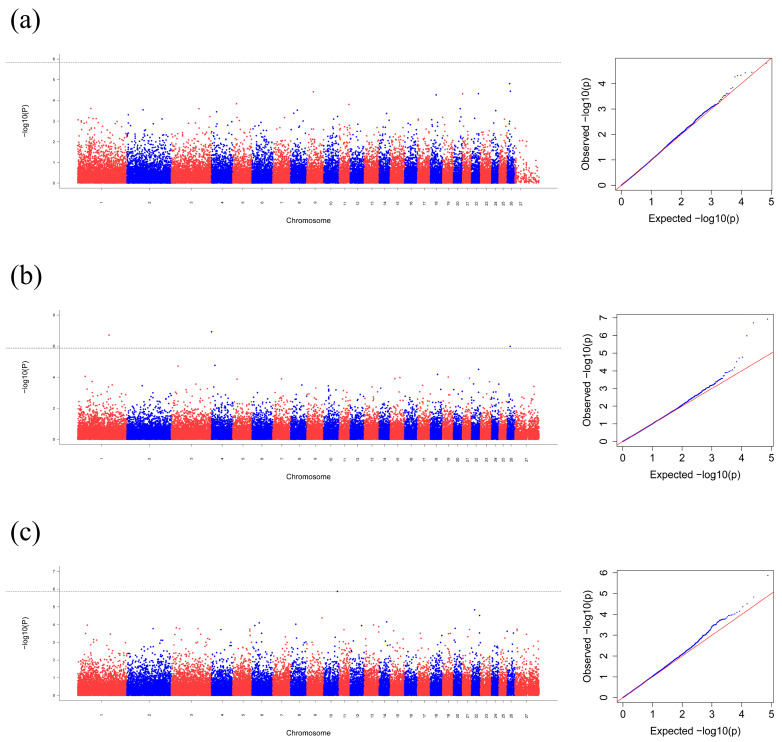
Manhattan plots and corresponding Q-Q plots genome-wide association signals across chromosomes (x-axis), with significance threshold lines; the y-axis represents the −log10 (*p*-value). In the Q_Q plots, the red line represents the expected value and the observed value (blue dot) deviates from this line. (**a**) Group 1, *p* = 1.48 × 10^−6^; (**b**) Group 2, *p* = 1.34 × 10^−6^; (**c**) Group 3, *p* ＝ 1.32 × 10^−6^.

An MT-GWAS was performed to identify gene associations with body size traits in Hulunbuir sheep, including chest girth, cannon circumference, hip width, body height, and body length. The quantile–quantile plots for body size traits are shown in Figure 2. The genome-wide significance level of 1.48 × 10^−6^, 1.34 × 10^−6^, 1.32 × 10^−6^ was calculated according to the number of 0.05/N (N represents the number of independent SNPs). The significance level of SNPs was identified based on their chromosomal locations (Figure 2).

Based on the calculated threshold, no significant SNPs were detected in either Group 1 or Group 3. In contrast, the analysis of body size traits in Group 2 revealed significant signals, with notable correlations observed on chromosomes 1, 4, and 26 (Figure 2b). As shown in Table 3, we identified 3 SNPs located in or close to 5 candidate genes related to body size traits in Hulunbuir sheep. These SNPs were located on chromosomes 1, 4, and 26; among them, solute carrier family 9 member C1 (*SLC9C1*) was located on chromosome 1; V-set and transmembrane domain containing 2A (*VSTM2A*) and LOC101105741 gene were located on chromosome 4; FSHD region gene 1 (*FRG1*) and *LOC105605168* gene were located on chromosome 26. 

### 3.3. Gene-Set Enrichment and Analysis

To achieve a more comprehensive insight into the biological functions associated with the candidate genes, the genes located closed to SNPs with *p*-values (*p* ≤ 0.05) for body size traits were analyzed by KEGG and GO enrichment analysis (Tables S1–S3); the results revealed significant enrichment in GO terms.

**Figure 3:**
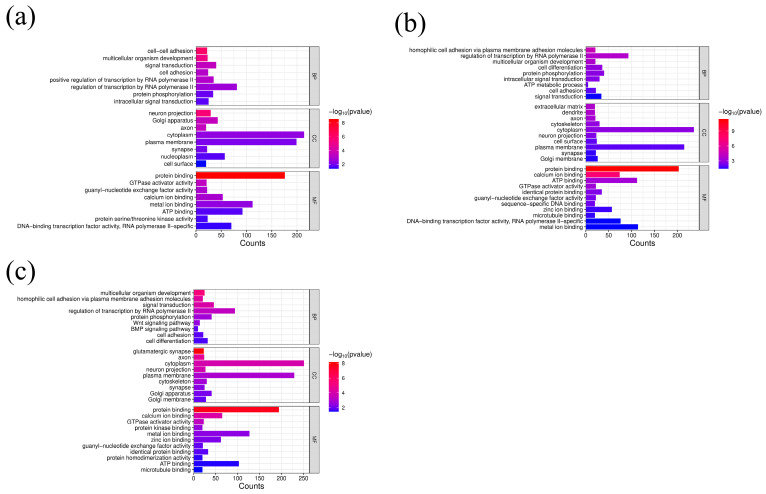
Gene Ontology terms enriched by genes associated with body size of three groups: (**a**) Group 1; (**b**) Group 2; (**c**) Group 3.

**Figure 4:**
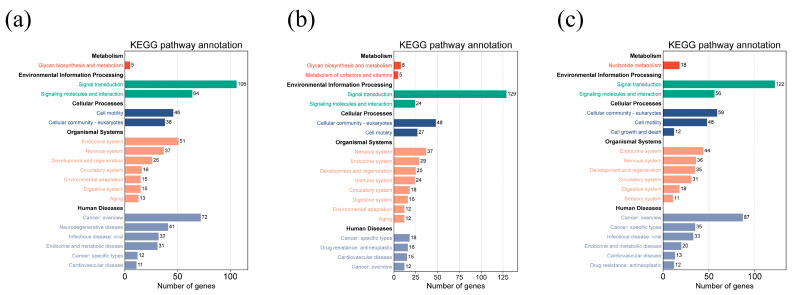
KEGG pathway enrichment analysis of the candidate genes associated with body size traits for three groups: (**a**) Group 1; (**b**) Group 2; (**c**) Group 3.

For Group 1, GO enrichment analysis revealed eight biological process terms, mainly including cell–cell adhesion, multicellular organism development, and signal transduction, etc., eight cellular component terms and eight molecular function terms. For Group 2, genes were primarily enriched in GO terms such as cytoplasm, plasma membrane, protein binding, and ATP binding. For Group 3, regulation of transcription by RNA polymerase II, cytoplasm, plasma membrane, and protein binding GO terms enriched a large number of genes (Figure 3). Moreover, pathways related to body size were identified by the KEGG analysis (Figure 4). The candidate genes are mainly enriched in pathways of metabolism, environmental information processing, cellular processes, organismal systems, and human diseases.

## 4. Discussion

Body size serves as a crucial growth performance indicator of individuals in the livestock industry. Currently, numerous studies have focused on body weight in sheep, whereas body size traits remain understudied. Our genome-wide association study identified 3 SNPs in Group 2 with significant associations to chest girth (CG), chest circumference (CC), hip width (HW), body height (BH), and body length (BL). These SNPs were mapped to genomic regions containing potential candidate genes with biological relevance to growth regulation. The SNP Chr4 g. 512090G>C localizes near *VSTM2A* and *LOC101105741*. *VSTM2A* encodes a secretory protein expressed during adipocyte differentiation, demonstrating elevated expression during early adipogenesis in vitro and adipose tissue development in vivo [23]. In mice, *VSTM2A* knockout reveals its critical role in lipid metabolism regulation, where deletion of *VSTM2A* promotes adipocyte hypertrophy and disrupts the homeostasis of glucose and lipid metabolism [24]. A recent study in chickens identified an ROH island spanning *VSTM2A* across 12 populations, with QTL mapping associating this region with body weight and carcass traits [25]. Our results are consistent with the aforementioned studies, indicating that the *VSTM2A* gene may have a potential impact on body size and weight traits in animals. However, its specific functions in livestock such as cattle and sheep remain unexplored. 

The SNP Chr1 g. 175232922G/A is located in the intron region of the *SLC9C1* gene. *SLC9C1* belongs to the *SLC9* gene family, which is responsible for encoding the ion transporter family of Na/H exchangers (NHEs) in eukaryotes. Studies have shown that *SLC9C1* is associated with sperm motility in both humans and mice [26]. Based on GWAS analysis, *SLC9C1* is associated with sperm traits in the Assaf sheep breed [27]. Our results contrast with a previous GWAS linking *SLC9C1* to sperm traits in Assaf sheep, the prior study emphasized reproductive rather than growth-related traits. This discrepancy may reflect breed-specific genetic architectures or phenotypic focus differences. 

In our study, another SNP (Chr26 g. 17254807G/T) was located near the *LOC105605168* and *FRG1* gene. The FSHD region gene 1 (*FRG1*) is the primary candidate gene for facioscapulohumeral muscular dystrophy (FSHD). Upregulating of FSHD may be associated with the disruption of skeletal muscles in the face, scapula, and humerus. Numerous studies have shown that *FRG1* is mainly related to muscle development and maintenance [28]. In a previous study, analysis of *FRG1* expression during vertebrate embryonic development using *Xenopus laevis* revealed that *FRG1* plays a crucial role in the development of tadpole musculature. Injection of *FRG1* morpholino disrupts the organization of the myotome and inhibits its growth. Elevated *FRG1* expression leads to abnormal formation of epaxial and hypaxial muscles. This indicates that normal *FRG1* expression is vital for proper muscle development [29]. We hypothesize that *FRG1* may influence body conformation through myogenic regulation, potentially affecting muscular development underlying growth traits.

Previous investigations on Hulunbuir sheep growth patterns have identified polymorphisms in IGF pathway components (*IGF1*, *IGF1R*). Three SNPs in *IGF1* were significantly correlated with four growth traits—chest girth at weaning (4 months), body length at 9 months, chest girth at 9 months, and average daily gain from 4 to 9 months (*p* < 0.05). For *IGF1R*, three SNPs and two haplotype blocks were significantly associated with 12 growth traits, including body height and body length at 4 months (*p* < 0.05) [30]. Additionally, two studies have tracked the body measurements of Hulunbuir sheep from birth to 16 months, identifying polymorphisms in the Somatostatin Receptor Subtype 1 (*SSTR1*) and *SSTR5* genes and their associations with growth traits [31,32]. These studies identified molecular markers that could serve as indicators for the growth traits of Hulunbuir sheep. While our current work did not examine these particular genes, the extended observation periods and comprehensive phenotyping in prior research highlight the importance of temporal dynamics in growth trait analyses.

In this study, functional enrichment analysis (KEGG/GO) of candidate genes revealed significant associations with fundamental cellular processes. As shown in Figure 3, cytoplasm, plasma membrane, protein binding, and ATP binding were enriched with candidate genes related to body size traits in all three groups. The cytoplasm is the main site of cellular metabolism and participates in a variety of biochemical reactions. It facilitates fundamental biochemical processes such as glycolysis—the foundational phase of aerobic respiration. In addition, the cytoplasm is involved in the transport of materials between the nucleus and the cytoplasm, such as the transport of ribosomal subunits, tRNA, and mRNA from the nucleus to the cytoplasm for protein synthesis. The plasma membrane serves as a critical interface between intracellular and extracellular environments while fulfilling essential biological functions including signal transduction, molecular transport regulation, and intercellular communication. The plasma membrane has a variety of receptor proteins, such as G protein-coupled receptors (GPCRs) and receptor tyrosine kinases (RTKs), which can recognize and bind to extracellular signaling molecules (such as hormones, neurotransmitters, etc.) and transmit signals into the cell, initiating downstream signaling pathways related to growth and development, such as the cAMP signaling pathway and the MAPK signaling pathway. The plasma membrane harbors specialized cell adhesion molecules that orchestrate both intercellular adhesion and cell-matrix anchoring, serving as molecular tethers for maintaining tissue architecture and mechanical stability. Studies have revealed that proteins in sheep embryonic skeletal muscle are significantly enriched in the pathways of protein binding, muscle contraction, and energy metabolism, while the growth and development of embryonic skeletal muscle play a crucial role in sheep muscle mass [33]. In Dorper and Hu sheep, it was found that genes were enriched in GO terms such as ATP binding and related pathways [34]. Similar to these two studies, we also found that protein binding and ATP binding GO terms were enriched with candidate genes related to body size traits in all three groups. Most genes were enriched in the signal transduction pathways within the environmental information processing category. The pathways under this category, such as the MAPK signaling pathway, mTOR signaling pathway, and Wnt signaling pathway, are all closely related to the development of skeletal muscle and energy metabolism, as well as the growth and development of the organism [35,36,37,38]. The observed enrichment patterns reinforce the evolutionary conservation of growth regulation networks while highlighting membrane-mediated signaling as a central regulatory layer in body size determination.

The limited number of genome-wide significant SNPs in our study may reflect inherent challenges in quantitative trait analysis. Despite more than 200 animals for livestock GWAS, a moderately sized cohort in this study, the sample size is still small compared to other species such as human [39]. Moreover, body size traits represent classic quantitative traits characterized by continuous phenotypic variation and significant environmental susceptibility. These traits typically exhibit polygenic inheritance involving multiple genetic loci [40]. Notable environmental sensitivity often results in genotype–environment interactions. Moreover, the effects of minor genes controlling the same quantitative trait are generally additive; this polygenic nature, combined with environmental modulation, presents significant challenges in identifying SNP loci and candidate genes. 

In this study, the genome-wide significance level of 1.48 × 10^−6^, 1.34 × 10^−6^, 1.32 × 10^−6^ was calculated according to the number of 0.05/N (N represents the number of independent SNPs). The threshold in Manhattan plots is −log10 (*p*-value), which is a strict standard of significance level. Previous studies have reported setting two thresholds for statistical significance at −log10 (5 × 10^−8^) and −log10 (1 × 10^−5^), representing genome-wide significance and suggestive significance, respectively [41,42]. This highlights the need for expanded sample sizes and meta-analyses to capture the complex genetic architecture underlying body size variation.

## 5. Conclusions

In conclusion, our study identified 3 significant SNPs associated with body size traits (CG, CC, HW, BH, and BL) using MT-GWAS in Hulunbuir sheep. A total of three candidate genes (*SLC9C1*, *VSTM2A*, *FRG1*) associated with body size traits were identified through GWAS analysis and KEGG pathway enrichment.

## Figures and Tables

**Table 1:** The grouping situation of Hulunbeier sheep.

Group	Age	The Number of Ewes	The Number of Rams
Group 1	4 months old	2	0
	6 months old	122	125
Group 2	12 months old	2	3
	24 months old	233	26
Group 3	36 months old	142	5
	48 months old	106	1
	60 months old	30	0
	70 months old	2	0

**Table 2:** Descriptive statistics of body size traits of Hulunbuir sheep.

Trait	Group	Mean	SD
Chest girth, cm	1	70.74	6.08
	2	85.89	8.25
	3	93.06	6.91
Cannon circumference, cm	1	7.25	0.53
	2	7.7	0.48
	3	7.71	0.46
Hip width, cm	1	15.91	1.92
	2	18.92	1.85
	3	19.59	1.63
Body height, cm	1	58.46	3.97
	2	61.96	6.87
	3	65.20	5.58
Body length, cm	1	62.47	5.41
	2	63.33	9.03
	3	63.82	7.53

**Table 3:** Information related to identified SNPs associated with body size traits.

Group	Chr.	Ref/Mut	Position (bp)	*p*-Value	Nearest Gene	Location
2	4	G/C	512,090	1.21 × 10^−7^	*VSTM2A*; *LOC101105741*	intergenic
2	1	G/A	175,232,922	1.95 × 10^−7^	*SLC9C1*	intron
2	26	G/T	17,254,807	1.05 × 10^−6^	*LOC105605168*; *FRG1*	intergenic

SNP, single nucleotide polymorphisms; Chr., chromosome; Ref/Mut, Reference/Mutation; Position, position (bp) on Oar_v4.0; Nearest Gene, nearest genes found on the NCBI database (Oar_v4.0); *p*-value, *p*-values calculated from the linear mixed model analysis.

## Data Availability

The original contributions presented in the study are included in the article/Supplementary Material, further inquiries can be directed to the corresponding authors.

## Supplementary Materials

The following supporting information can be downloaded at: https://www.mdpi.com/article/10.3390/genes16040410/s1, Tables S1–S3: Gene ontology terms and KEGG pathways for body size traits of Group 1–3 were significantly enriched (*p* ≤ 0.05).

## References

[1] Shi Y., Qi Y., Liu Y., Rong Y., Ao X., Zhang M., Xia Q., Zhang Y., Wang R. (2024). Study of the Influence of Non-Genetic Factors on the Growth and Development Traits and Cashmere Production Traits of Inner Mongolia White Cashmere Goats (Erlangshan Type). Vet. Sci..

[2] Zhang X., Li G., Li F., Zhang D., Yuan L., Zhao Y., Zhang Y., Li X., Song Q., Wang W. (2023). Effect of feed efficiency on growth performance, body composition, and fat deposition in growing Hu lambs. Anim. Biotechnol..

[3] Kemper K.E., Visscher P.M., Goddard M.E. (2012). Genetic architecture of body size in mammals. Genome Biol..

[4] Ahmad S.F., Khan N.N., Ganai N.A., Shanaz S., Rather M.A., Alam S. (2021). Multivariate quantitative genetic analysis of body weight traits in Corriedale sheep. Trop. Anim. Health Prod..

[5] Erdenee S., Akhatayeva Z., Pan C., Cai Y., Xu H., Chen H., Lan X. (2021). An insertion/deletion within the CREB1 gene identified using the RNA-sequencing is associated with sheep body morphometric traits. Gene.

[6] Kijas J.W., Townley D., Dalrymple B.P., Heaton M.P., Maddox J.F., McGrath A., Wilson P., Ingersoll R.G., McCulloch R., McWilliam S. (2009). A genome wide survey of SNP variation reveals the genetic structure of sheep breeds. PLoS ONE.

[7] Tosser-Klopp G., Bardou P., Bouchez O., Cabau C., Crooijmans R., Dong Y., Donnadieu-Tonon C., Eggen A., Heuven H.C., Jamli S. (2014). Design and characterization of a 52K SNP chip for goats. PLoS ONE.

[8] Zhu C., Fan H., Yuan Z., Hu S., Ma X., Xuan J., Wang H., Zhang L., Wei C., Zhang Q. (2016). Genome-wide detection of CNVs in Chinese indigenous sheep with different types of tails using ovine high-density 600K SNP arrays. Sci. Rep..

[9] Becker D., Tetens J., Brunner A., Bürstel D., Ganter M., Kijas J., Drögemüller C. (2010). Microphthalmia in Texel sheep is associated with a missense mutation in the paired-like homeodomain 3 (PITX3) gene. PLoS ONE.

[10] Stephens M. (2013). A unified framework for association analysis with multiple related phenotypes. PLoS ONE.

[11] Bolormaa S., Pryce J.E., Reverter A., Zhang Y., Barendse W., Kemper K., Tier B., Savin K., Hayes B.J., Goddard M.E. (2014). A multi-trait, meta-analysis for detecting pleiotropic polymorphisms for stature, fatness and reproduction in beef cattle. PLoS Genet..

[12] Xu H., Zhang X., Zang R., Cai Y., Cao X., Yang J., Li J., Lan X., Wu J. (2019). Genetic variations in the sheep SIRT7 gene and their correlation with body size traits. Arch. Anim. Breed..

[13] He S., Di J., Han B., Chen L., Liu M., Li W. (2020). Genome-Wide Scan for Runs of Homozygosity Identifies Candidate Genes Related to Economically Important Traits in Chinese Merino. Animals.

[14] Tao L., Liu Y.F., Zhang H., Li H.Z., Zhao F.P., Wang F.Y., Zhang R.S., Di R., Chu M.X. (2021). Genome-wide association study and inbreeding depression on body size traits in Qira black sheep (*Ovis aries*). Anim. Genet..

[15] Jiang J., Cao Y., Shan H., Wu J., Song X., Jiang Y. (2021). The GWAS Analysis of Body Size and Population Verification of Related SNPs in Hu Sheep. Front. Genet..

[16] Tuersuntuoheti M., Zhang J., Zhou W., Zhang C.L., Liu C., Chang Q., Liu S. (2023). Exploring the growth trait molecular markers in two sheep breeds based on Genome-wide association analysis. PLoS ONE.

[17] Posbergh C.J., Huson H.J. (2021). All sheeps and sizes: A genetic investigation of mature body size across sheep breeds reveals a polygenic nature. Anim. Genet..

[18] Li X., He S.G., Li W.R., Luo L.Y., Yan Z., Mo D.X., Wan X., Lv F.H., Yang J., Xu Y.X. (2022). Genomic analyses of wild argali, domestic sheep, and their hybrids provide insights into chromosome evolution, phenotypic variation, and germplasm innovation. Genome Res..

[19] Li J., Zhang N., Tian X., Tian P., Yang C., Chen J., Yan H., Duan C., Guo Y., Liu Y. (2021). Construction of Growth Model of Mutton Sheep and Prediction of Growth Performance. Chin. J. Anim. Nutr..

[20] Guo Y., Bai F., Wang J., Fu S., Zhang Y., Liu X., Zhang Z., Shao J., Li R., Wang F. (2023). Design and characterization of a high-resolution multiple-SNP capture array by target sequencing for sheep. J. Anim. Sci..

[21] Purcell S., Neale B., Todd-Brown K., Thomas L., Ferreira M.A., Bender D., Maller J., Sklar P., de Bakker P.I., Daly M.J. (2007). PLINK: A tool set for whole-genome association and population-based linkage analyses. Am. J. Hum. Genet..

[22] Zhou X., Stephens M. (2014). Efficient multivariate linear mixed model algorithms for genome-wide association studies. Nat. Methods.

[23] Secco B., Camiré É., Brière M.A., Caron A., Billong A., Gélinas Y., Lemay A.M., Tharp K.M., Lee P.L., Gobeil S. (2017). Amplification of Adipogenic Commitment by VSTM2A. Cell Rep..

[24] Al Dow M., Secco B., Mouchiroud M., Rochette M., Gilio G.R., Massicard M., Hardy M., Gélinas Y., Festuccia W.T., Morissette M.C. (2025). Loss of VSTM2A promotes adipocyte hypertrophy and disrupts metabolic homeostasis. Obesity.

[25] Tan X., Liu L., Dong J., Huang M., Zhang J., Li Q., Wang H., Bai L., Cui M., Zhou Z. (2024). Genome-wide detections for runs of homozygosity and selective signatures reveal novel candidate genes under domestication in chickens. BMC Genom..

[26] Cavarocchi E., Whitfield M., Chargui A., Stouvenel L., Lorès P., Coutton C., Arnoult C., Santulli P., Patrat C., Thierry-Mieg N. (2021). The sodium/proton exchanger SLC9C1 (sNHE) is essential for human sperm motility and fertility. Clin. Genet..

[27] Serrano M., Ramón M., Calvo J.H., Jiménez M., Freire F., Vázquez J.M., Arranz J.J. (2021). Genome-wide association studies for sperm traits in Assaf sheep breed. Animal.

[28] Hansda A.K., Tiwari A., Dixit M. (2017). Current status and future prospect of FSHD region gene 1. J. Biosci..

[29] Hanel M.L., Wuebbles R.D., Jones P.L. (2009). Muscular dystrophy candidate gene FRG1 is critical for muscle development. Dev. Dyn..

[30] Ding N., Tian D., Li X., Zhang Z., Tian F., Liu S., Han B., Liu D., Zhao K. (2022). Genetic Polymorphisms of IGF1 and IGF1R Genes and Their Effects on Growth Traits in Hulun Buir Sheep. Genes.

[31] Li X., Ding N., Zhang Z., Tian D., Han B., Liu S., Liu D., Tian F., Zhao K. (2021). Identification of Somatostatin Receptor Subtype 1 (SSTR1) Gene Polymorphism and Their Association with Growth Traits in Hulun Buir Sheep. Genes.

[32] Li X., Ding N., Zhang Z., Tian D., Han B., Liu D., Liu S., Tian F., Fu D., Song X. (2022). Identification of SSTR5 Gene Polymorphisms and Their Association With Growth Traits in Hulun Buir Sheep. Front. Genet..

[33] Wang X., Shi T., Zhao Z., Hou H., Zhang L. (2020). Proteomic analyses of sheep (*Ovis aries*) embryonic skeletal muscle. Sci. Rep..

[34] Lv X., Chen W., Wang S., Cao X., Yuan Z., Getachew T., Mwacharo J.M., Haile A., Sun W. (2023). Whole-genome resequencing of Dorper and Hu sheep to reveal selection signatures associated with important traits. Anim. Biotechnol..

[35] Lou M., Zhang S., Yang W., Li S., Cao H., Zhang Z., Ling Y. (2025). Transcriptome analysis revealed the mechanism of skeletal muscle growth and development in different hybrid sheep. Anim. Biosci..

[36] Verbrugge S.A.J., Schönfelder M., Becker L., Yaghoob Nezhad F., Hrabě de Angelis M., Wackerhage H. (2018). Genes Whose Gain or Loss-Of-Function Increases Skeletal Muscle Mass in Mice: A Systematic Literature Review. Front. Physiol..

[37] Samereh S., Hajarian H., Karamishabankareh H., Soltani L., Foroutanifar S. (2021). Effects of different concentrations of Chir98014 as an activator of Wnt/beta-catenin signaling pathway on oocyte in-vitro maturation and subsequent embryonic development in Sanjabi ewes. Reprod. Domest. Anim..

[38] Chen Q., Bao J.J., Zhang H.C., Huang C., Zhao Q., Pu Y.B., Jiang L., Hosseiny A., Ibrahim M., Hussain T. (2024). LncRNA GTL2 regulates myoblast proliferation and differentiation via the PKA-CREB pathway in Duolang sheep. Zool. Res..

[39] Yengo L., Sidorenko J., Kemper K.E., Zheng Z., Wood A.R., Weedon M.N., Frayling T.M., Hirschhorn J., Yang J., Visscher P.M. (2018). Meta-analysis of genome-wide association studies for height and body mass index in ∼700000 individuals of European ancestry. Hum. Mol. Genet..

[40] Li M., Zhu L., Li X., Shuai S., Teng X., Xiao H., Li Q., Chen L., Guo Y., Wang J. (2008). Expression profiling analysis for genes related to meat quality and carcass traits during postnatal development of backfat in two pig breeds. Sci. China C Life Sci..

[41] Schaid D.J., Chen W., Larson N.B. (2018). From genome-wide associations to candidate causal variants by statistical fine-mapping. Nat. Rev. Genet..

[42] Yang T., Wang M., Liu Y., Li Y., Feng M., Zhao C. (2024). A mutation in POLR2A gene associated with body size traits in Dezhou donkeys revealed with GWAS. J. Anim. Sci..

